# Characterizing modifications to a comparative effectiveness research study: the OPTIMIZE trial—using the Framework for Reporting Adaptations and Modifications to Evidence-based Interventions (FRAME)

**DOI:** 10.1186/s13063-023-07150-1

**Published:** 2023-02-23

**Authors:** Julie M. Fritz, Tom Greene, Gerard P. Brennan, Kate Minick, Elizabeth Lane, Stephen T. Wegener, Richard L. Skolasky

**Affiliations:** 1 Department of Physical Therapy & Athletic Training, University of Utah, 383 Colorow Drive, Room 391, Salt Lake City, UT 84108 USA; 2 Department of Population Health Sciences, University of Utah, Salt Lake City, UT USA; 3 Rehabilitation Services, Intermountain Healthcare, Salt Lake City, UT USA; 4 Department of Physical Medicine and Rehabilitation, Johns Hopkins University, Baltimore, MD USA; 5 Department of Orthopaedic Surgery, Johns Hopkins University, Baltimore, MD USA

**Keywords:** Comparative effectiveness research, Pragmatic clinical trial, Low back pain, Modification, Fidelity, Implementation

## Abstract

**Background:**

The OPTIMIZE trial is a multi-site, comparative effectiveness research (CER) study that uses a Sequential Multiple Assessment Randomized Trial (SMART) designed to examine the effectiveness of complex health interventions (cognitive behavioral therapy, physical therapy, and mindfulness) for adults with chronic low back pain. Modifications are anticipated when implementing complex interventions in CER. Disruptions due to COVID have created unanticipated challenges also requiring modifications. Recent methodologic standards for CER studies emphasize that fully characterizing modifications made is necessary to interpret and implement trial results. The purpose of this paper is to outline the modifications made to the OPTIMIZE trial using the Framework for Reporting Adaptations and Modifications to Evidence-Based Interventions (FRAME) to characterize modifications to the OPTIMIZE trial in response to the COVID pandemic and other challenges encountered.

**Methods:**

The FRAME outlines a strategy to identify and report modifications to evidence-based interventions or implementation strategies, whether planned or unplanned. We use the FRAME to characterize the process used to modify the aspects of the OPTIMIZE trial. Modifications were made to improve lower-than-anticipated rates of treatment initiation and COVID-related restrictions. Contextual modifications were made to permit telehealth delivery of treatments originally designed for in-person delivery. Training modifications were made with study personnel to provide more detailed information to potential participants, use motivational interviewing communication techniques to clarify potential participants’ motivation and possible barriers to initiating treatment, and provide greater assistance with scheduling of assigned treatments.

**Results:**

Modifications were developed with input from the trial’s patient and stakeholder advisory panels. The goals of the modifications were to improve trial feasibility without compromising the interventions’ core functions. Modifications were approved by the study funder and the trial steering committee.

**Conclusions:**

Full and transparent reporting of modifications to clinical trials, whether planned or unplanned, is critical for interpreting the trial’s eventual results and considering future implementation efforts.

**Trial registration:**

ClinicalTrials.gov NCT03859713. Registered on March 1, 2019

## Background

**Fig. 1:**
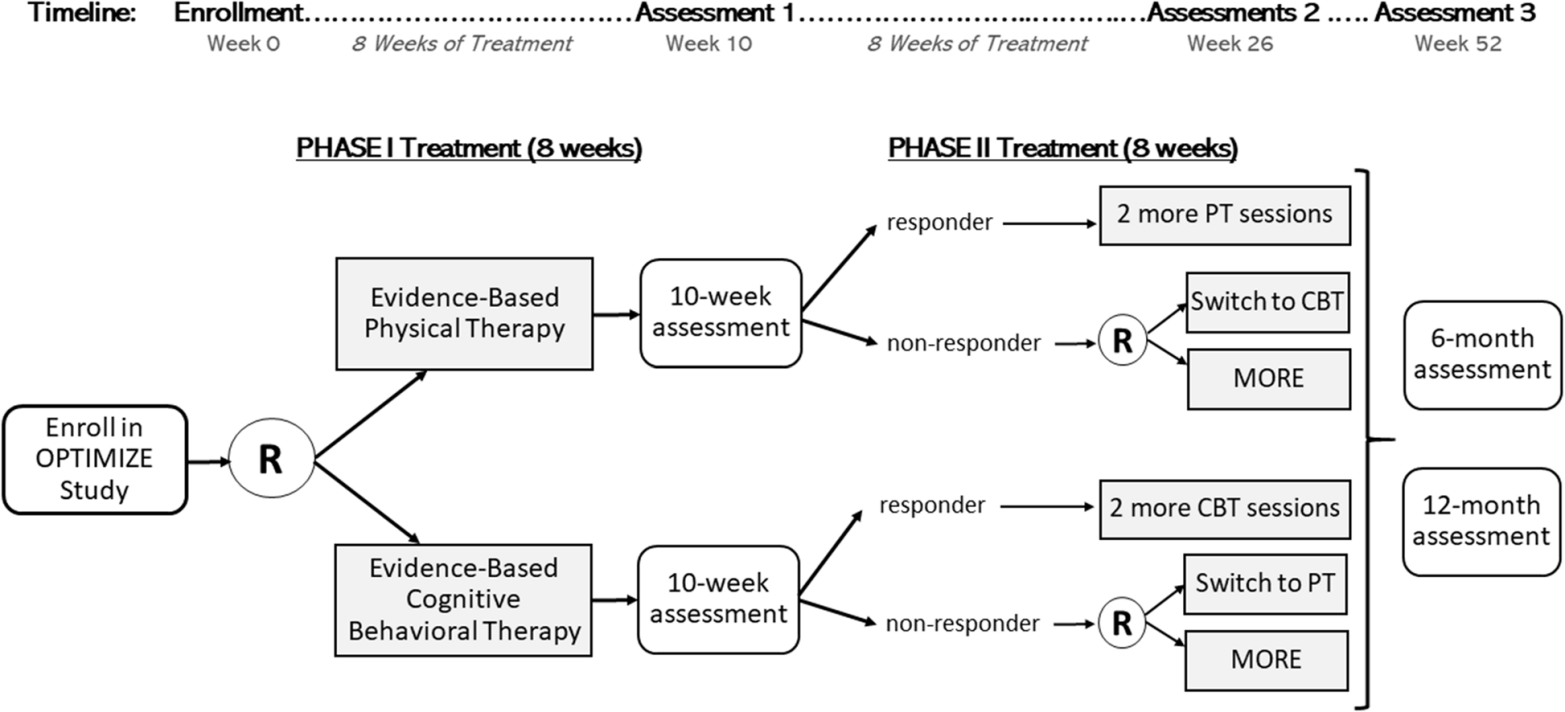
Study design for the OPTIMIZE study, a Sequential Multiple Assessment Randomized Trial. CBT, cognitive behavioral therapy; MORE, Mindfulness-Oriented Recovery Enhancement; PT, physical therapy; R, randomize

The OPTIMIZE trial is a comparative effectiveness, multi-site randomized clinical trial with a Sequential Multiple Assessment Randomized Trial (SMART) design (Fig. 1) [1]. The trial is investigating three evidence-based, non-pharmacologic interventions for patients with chronic low back pain (cLBP), cognitive behavioral therapy (CBT), physical therapy (PT), and a mindfulness program (Mindfulness-Oriented Recovery Enhancement (MORE)). The trial protocol is published [1]. As a comparative effectiveness research (CER) trial funded by the Patient-Centered Outcomes Research Institute (PCORI), the long-term goal of the OPTIMIZE trial is to provide end-users, patients, and providers with information to inform the selection of interventions that are most likely to lead to better health outcomes for individual patients and, if the first attempted treatment is ineffective, to inform the sequence of treatments most likely to optimize outcomes [2].

Each treatment studied in the OPTIMIZE trial represents a complex health intervention (CHI) because each comprises multiple, interactive components that are implemented with flexibility in routine care and require behavioral changes by the person receiving the interventions [3, 4]. Implementing CHIs in CER studies is expected to involve modifications to adapt to the needs of local settings, respond to contextual factors, and reflect real-world practices [5]. A modification represents a planned or unplanned change to an evidence-based intervention or its delivery in an attempt to improve the fit, fidelity, or effectiveness of the intervention [6]. While it is desirable to anticipate and plan for modifications prior to beginning a CER trial, unanticipated modifications are often necessary to respond to unforeseen challenges [7]. The COVID-19 pandemic has required many modifications in real-world care delivery settings and specifically for the delivery of nonpharmacologic interventions for LBP such as those included in the OPTIMIZE trial [8].

Modifications to evidence-based interventions, particularly those that occur during the execution of a clinical trial, should be comprehensively characterized and documented because of the impact on the interpretation of the study’s findings and future implementation efforts [9]. Recent methodology standards from PCORI to improve CER involving CHIs support the need to monitor and describe modifications [7]. Several frameworks have been developed to monitor and characterize modifications to pragmatic and CER trials [10]. The Framework for Reporting Adaptations and Modifications-Enhanced (FRAME) outlines a strategy to identify and report modifications to evidence-based interventions or implementation strategies, whether planned or unplanned [11, 12]. The purpose of this paper is to use the FRAME model to characterize the modifications made to the OPTIMIZE trial in response to the COVID pandemic and other challenges encountered in the participating health care delivery systems.

## Methods

### The OPTIMIZE trial

The OPTIMIZE trial is being conducted in three health care systems in two geographic locations: Salt Lake City, UT, and Baltimore, MD [1]. The design is described in Fig. 1. Following informed consent and baseline assessment, participants are randomized within 14 days to receive 8 weeks of phase I treatment with either PT or CBT. The goal is to initiate phase I treatment within 30 days of randomization although this is not always possible based on provider availability. These treatments were selected as phase I comparators because they are evidence-based [13] and is commonly used [14–16]. However, their effectiveness has not been previously compared for patients with cLBP. Approximately 10 weeks after enrollment, participants who have not adequately responded to their initial treatment assignment (defined as a 50% reduction in pain-related disability) are randomly assigned to a phase II treatment of either (1) the alternate phase I treatment or (2) MORE and receive another 8 weeks of the assigned phase II treatment. The rationale for phase II is to permit evaluation of adaptive interventions which reflect real-world clinical circumstances in which patients are likely to try new treatments if their initial treatment is not effective. Outcome assessments occur 10, 26, and 52 weeks following randomization.

**Fig. 2:**
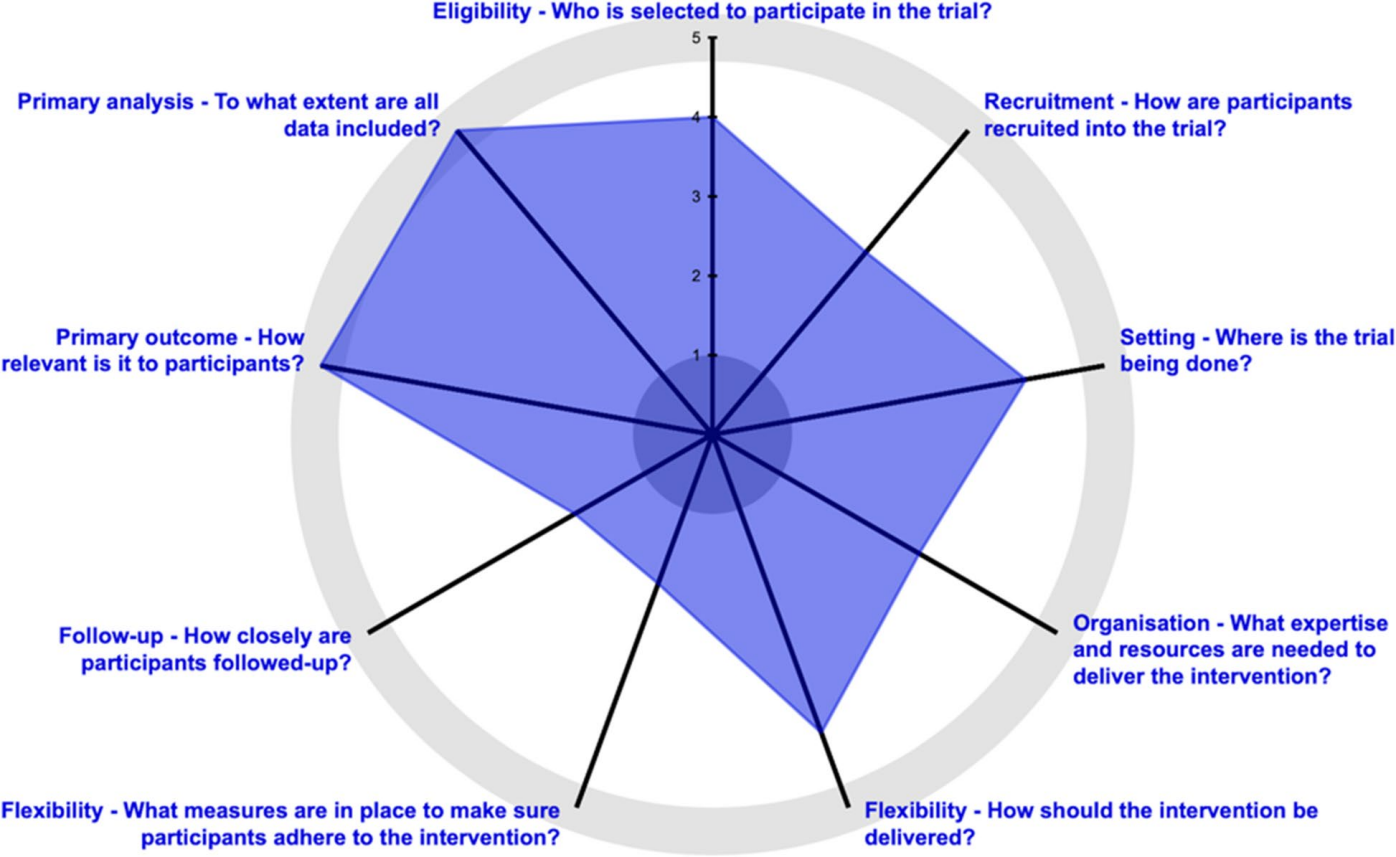
PRECIS-2 scoring wheel for the OPTIMIZE study. This is a visual representation of pragmatism of the trial on the explanatory–pragmatic continuum from the original study protocol publication [1]

As a CER study, OPTIMIZE is intended to reflect and inform real-world care delivery using mostly pragmatic methods [17]. In planning the trial, the OPTIMIZE team outlined the levels of pragmatism across the nine domains of the Pragmatic-Explanatory Continuum Indicator Summary (PRECIS-2) [18]. As outlined in the PRECIS-2 wheel included in the original study protocol (Fig. 2), the trial was designed with a high degree of pragmatism in most domains including the primary outcome, settings, eligibility criteria, and flexibility for providers delivering the interventions [1]. Less pragmatism was used in the domains of recruitment and flexibility in participant adherence. This was done because barriers to engaging in CHIs such as those in the OPTIMIZE trial emerged in our conversations with patients and are documented in the literature. Rates of initiation of physical and behavioral health therapies can be low among referred patients [19, 20]. High out-of-pocket expenses and lack of insurance coverage are barriers to initiating and adhering to CHIs for chronic pain [21, 22]. The OPTIMIZE team anticipated that without assistance, treatment initiation rates could preclude rigorous evaluation of the study aims if too few participants began treatment. To counter this concern, study personnel who screen and consent participants were trained to provide information about the possibility that they may have out-of-pocket financial obligations and should check with their insurance provider, and after enrollment, the study staff provided information about the nearest clinic with a provider trained in OPTIMIZE interventions [1]. We considered these methods less than fully pragmatic (Fig. 2).

### Fidelity monitoring for OPTIMIZE

Monitoring fidelity in the CER and pragmatic trials, particularly those involving CHIs, is an important consideration to accurately interpret the trial’s findings [23, 24]. Without strategies to monitor fidelity, it is difficult to discern whether a trial’s findings are attributable to the intervention being studied or the extent to which participants were able to actually engage with the intervention, particularly when no difference is detected [25]. Fidelity monitoring is also an important consideration during the conduct of a CER study. Although a greater degree of variability in the decision of participants to initiate and persist in treatment is expected in CER and pragmatic studies, extremely low levels of uptake will not allow researchers to address a study’s aims related to a treatment’s effectiveness and should be monitored by investigators and as part of data and safety monitoring procedures [26].

Prior to beginning enrollment, the OPTIMIZE team established plans to monitor barriers to implementation across sites using the Consolidated Framework for Implementation Research (CFIR) model [27]. The CFIR includes domains related to the characteristics of the interventions and the implementation process. Monitoring fidelity in the implementation of CHIs requires distinguishing between core functions and forms of each intervention [28]. Core functions represent the fundamental purpose or desired effects of a CHI while forms describe the activities used to carry out a CHI’s core functions [29]. In CER studies, PCORI methodologic standards specify that modifications should preserve the core functions to ensure the same intervention is studied across sites, whereas modifications to forms are expected to occur [7]. The OPTIMIZE team set up fidelity checklists in the electronic health record (EHR) of the participating health care systems to monitor fidelity to the core functions of each CHI which included treatment initiation as well as specific components provided by therapists once treatment was initiated [1].

### The FRAME framework

**Fig. 3:**
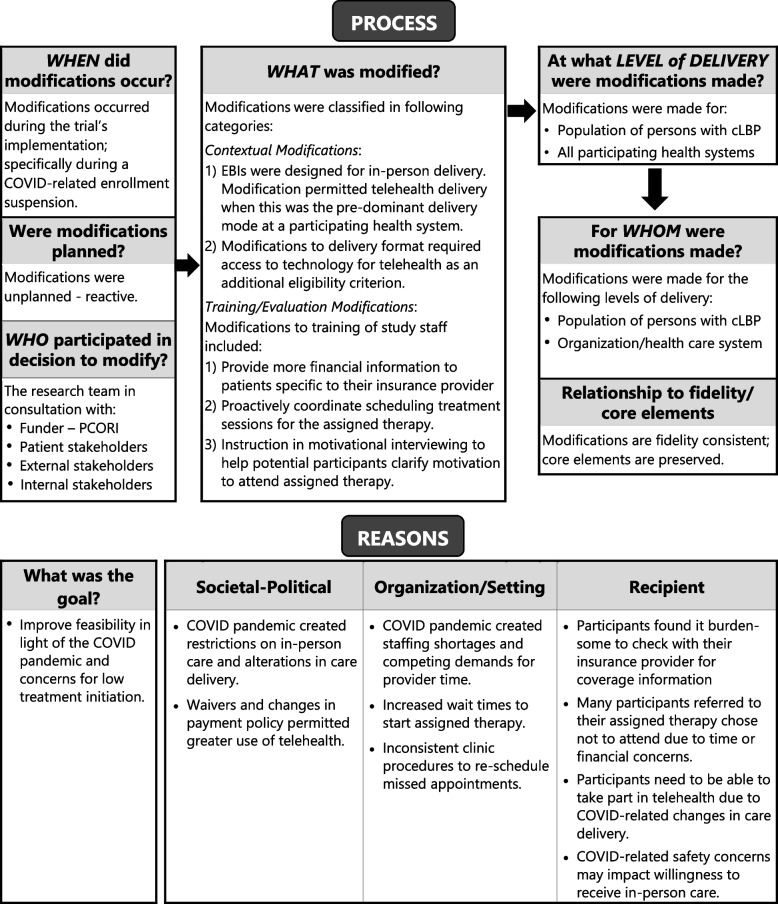
Modifications to the OPTIMIZE trial characterized using the FRAME [11]

The FRAME was developed to help study teams identify and fully characterize modifications to evidence-based interventions and the strategies used to implement them [11, 30]. FRAME outlines the components of modifications to report (1) when modifications were made; (2) whether the modification was planned or unplanned; (3) who participated in the decisions about modifications, (4) what was modified, delivery level of modifications; (6) the relationship between modifications and fidelity; and (7) rationale for modifications [11]. Modifications to the OPTIMIZE trial were assessed across these components (Fig. 3).

## Results

### When modifications were made

Modifications were made during the implementation of the OPTIMIZE trial. The OPTIMIZE trial initiated enrollment in March 2019. In March 2020, with the onset of the COVID-19 pandemic, the trial suspended new enrollments across all sites due to health system restrictions on in-person care and non-essential clinical research activities. A total of 181 participants had enrolled across sites at the time of suspension. Just prior to the onset of COVID, in February 2020, the trial’s Data and Safety Monitoring Board met. Assessment of the overall treatment initiation rate was part of the open report for the board meeting. Initiation rates by treatment group were part of the closed report from which the investigators remained blinded. At that time, the treatment initiation rate for phase I was 65%, and 43% for phase II. These findings prompted the study team to develop a modification strategy to increase treatment initiation across phases. Before the modifications could be implemented, the COVID pandemic suspended enrollment in the trial.

### Whether modifications were planned or unplanned

Modifications made to the OPTIMIZE trial were reactive to the challenges of low treatment initiation and COVID-related impacts on in-person care delivery. Although treatment initiation was an aspect of implementing the trial that was designed to be less than fully pragmatic [18], the modifications undertaken were not planned prior to beginning the trial.

### Who participated in the decisions about modifications

The decision-making process to determine the modifications to the OPTIMIZE trial involved the study team, the study’s patient advisory and external stakeholder panels, and participants enrolled in the OPTIMIZE study prior to the pandemic. Modifications were also discussed and eventually approved by representatives from the funder (PCORI). Modifications were also approved by the study’s single Institutional Review Board.

The study team met with the patient advisory panel just prior to the COVID pandemic to help identify reasons for lower-than-expected treatment initiation. Feedback from the patient advisors was that financial concerns may be a reason, and the panel discussed how best to explain the treatment options to participants.

The onset of COVID suspended enrollment due to health system restrictions on in-person care and non-essential clinical research. The initial determination of the study team in consultation with representatives from the funder was to wait to resume enrollment until the CHIs could be delivered in person as originally intended. This decision was based on safety concerns associated with in-person care and the potential differential impact of telehealth delivery on the study CHIs, which could negatively influence clinical equipoise because the PT intervention involves physical touch while CBT and MORE do not.

As the pandemic persisted, the OPTIMIZE team identified potential modifications that would permit re-starting enrollment. The options were presented to the study’s external advisory panel. This panel included stakeholders representing the perspectives of health systems, payers, and provider groups, as well as persons living with chronic pain. The discussion focused on concerns about the acceptability to patients and effectiveness of delivering PT via telehealth, the potential adverse impact on the ability to include persons with lower economic resources and access to technology if the ability to participate in telehealth were an eligibility requirement, and the likelihood that waivers implemented by insurance providers to permit reimbursement of PT provided via telehealth would persist.

Some of the issues discussed with the external advisory panel were further informed by a survey of participants enrolled in the OPTIMIZE trial prior to the COVID enrollment suspension. The survey was conducted in September to October of 2020. Among survey respondents, 9% indicated that access to technology would have been a barrier to telehealth participation, while 43% indicated they would be unwilling to participate in PT delivered via telehealth, compared to 24% indicating unwillingness to participate in telehealth behavioral health sessions [31]. These findings lessened concerns about technology access but supported the concern that shifting to telehealth delivery would differently impact the OPTIMIZE interventions.

### What was modified

FRAME outlines three classifications of modifications [12]. Content modifications change the core functions of a CHI. Contextual modifications change the manner of delivering CHI content such as changes to the format, settings, personnel delivering, or the population receiving the CHI. Training and evaluation modifications change how the staff or research personnel are trained or the way in which CHIs are evaluated. None of the modifications to the OPTIMIZE trial was content modifications. Modifications were made in the contextual and training and evaluation classifications to address concerns for low treatment initiation and COVID-related changes to in-person delivery. These modifications are described below.

#### Contextual modifications

The modification to resolve the issue of changes to in-person delivery was to deliver CHIs to OPTIMIZE participants using the delivery format (in-person or telehealth) that was predominant in the health system at the time of enrollment. For example, a patient enrolled and randomized to PT in phase I would be recommended to attend in-person PT if at least half of the PT sessions were being provided in person in the health care system at the time. The same modification applied to the other CHIs in the study and to phase II assignments. Similar to many health care delivery settings, after the initial weeks of the COVID pandemic, behavioral health providers, who deliver CBT and MORE, continued to predominantly use telehealth, while PT providers largely resumed in-person care [32, 33]. Therefore, since the time this modification was made, all participating health care systems have been providing PT predominantly in-person and behavioral health using telehealth. This modification preserved the core functions of the OPTIMIZE CHIs while also providing a strategy to adapt to any future disruptions or changes to the delivery format that may occur.

The modification to allow telehealth delivery of some CHIs required the ability to participate in telehealth be added as a criterion for eligibility to participate in the OPTIMIZE study. The number of persons unable to participate due to this criterion was added to the study’s tracking of screening procedures so that the number of persons unable to participate for this reason is documented. The study’s single IRB approved these changes to permit the resumption of enrollment into the study.

#### Training and evaluation modifications

Modifications to address low treatment initiation focused on additional training of the study staff responsible for screening and consenting OPTIMIZE participants. These modifications are outlined below.

1. The first modification was to be more attentive to financial implications for potential participants. Pragmatic and CER studies typically do not reimburse out-of-pocket expenses encountered by participants in order to reflect real-world circumstances [34]. At the start of the study, the staff notified potential participants that they may incur out-of-pocket expenses based on their insurer’s coverage, deductible and co-payment policies, and encouraged individuals to check with their insurer for more information. We modified this process to provide more specific information on likely co-pay amounts for a particular insurer. We also identified insurers at each site that denied covered to one of the study’s CHIs and stopped efforts to enroll these persons. The staff document reasons that individuals provide for not participating in the OPTIMIZE study to allow the impact of these efforts on enrollment to be tracked.
2. We modified the role the study staff played in scheduling treatments session with participants. At the start of the study, the OPTIMIZE staff provided participants with contact information for providers but left the responsibility for scheduling to the participant. If scheduled appointments were missed, it was left to the clinic and their usual procedures to contact participants to re-schedule. The onset of COVID exacerbated scheduling challenges due to provider shortages and extended wait times for appointments. Recognition that the overall complexity of scheduling visits was a barrier to treatment initiation and persistence, the OPTIMIZE team modified the training of study staff to adopt a navigator role. Specific changes made included having staff directly connect participants to clinics to schedule the initial session. The staff track the participant’s attendance at the initial session and follow-up with the participant to assist with re-scheduling. When participants encounter long wait times to initiate treatment, the staff contact them intermittently to provide reminders.
3. We modified the staff training in communication with potential participants. The trans-theoretical model of change indicates that some persons may fail to initiate or persist in a CHI based on a lack of readiness or ambivalence about making this type of behavior change [35, 36]. Motivational interviewing (MI) is a collaborative communication approach to help clarify and strengthen a person’s motivation and commitment to change by exploring and resolving ambivalence [37]. Prior to resuming enrollment following the COVID-related suspension, the OPTIMIZE team provided study staff with brief MI training to help potential participants clarify their level of motivation to attend their assigned CHI should they choose to participate. The research team has experience in training staff to implement MI techniques to increase the likelihood of initiating treatment [38]. This experience was adapted to explore a person’s confidence in initiating treatment and, through identifying potential barriers and facilitators, to resolve any ambivalence. The staff participated in two web-based video-enabled training sessions led by study investigators (STW and RLS) that focused on MI principles of open-ended communication, affirmations, rolling with resistance, and summarization. These principles were practiced among the staff in separate telephone-based dyads to simulate talking with potential participants during the recruitment process. For example, the staff ask a potential participant about their confidence in their ability to attend weekly sessions should they choose to participate and to rate their confidence on a scale from 0 (not at all confident) to 10 (completely confident). The staff then ask the potential participant about their reasoning for their confidence rating. The goal of this process is not necessarily to have more persons enroll in the study but to increase awareness of what participation involves and enroll persons with a higher likelihood of initiating and persisting through two treatment phases.

### Delivery level of modifications

Modifications to the OPTIMIZE trial were applied across the participating health care systems. While the determination to provide a particular CHI in-person or via telehealth was based on local conditions, the principle governing the modification (i.e., to provide care in the format that was in predominant use at the time) was consistent across systems. All potential participants were screened at all sites using the training and evaluation modifications described above.

### Fidelity and modifications

It is important to consider the relationship between modifications and preservation of the core functions of a CHI [6]. Modifications that adjust forms but preserve core functions of CHIs are categorized as fidelity-consistent, while modifications that alter core functions are considered fidelity-inconsistent [29]. Concerns that permitting telehealth delivery of the PT intervention would be fidelity-inconsistent was a source of considerable debate among the study team, representatives of the funder, and patient and external stakeholders. Physical touch as part of manual therapy was identified by the study team as a core function of evidence-based PT prior to beginning the study [39]. Physical touch was not identified as a core function for CBT and MORE and existing literature support equivalence of in-person or telehealth delivery of these interventions [40, 41]. The modification to provide the CHIs using the predominant format in the health system at the time of recruitment has proven to be a fidelity-consistent modification because the application of this modification has meant that the PT intervention is provided in-person, while CBT and MORE interventions have been provided using telehealth at all sites since the resumption of enrollment. Modifications to the training of the study staff are considered fidelity-consistent as they do not impact CHI core functions.

### Rationale for modifications

Clarifying the rationale for modifications requires specifying both the goals and reasons that modifications are made [11]. At the beginning of the pandemic, the OPTIMIZE team established four principles that had to be balanced when considering potential modifications: (1) maintain the safety of research participants, study personnel, providers, and stakeholders; (2) maintain patient-centeredness in the research; (3) maintain scientific rigor in the research and fidelity to the study’s original research questions; and (4) abide by all safety and public health policies at the health system, local, state, and federal levels. The modifications made were guided by these principles and were made with the goal of improving the feasibility of the study in light of both the COVID pandemic and concerns about low treatment initiation.

The reasons underlying the modifications made existed at several levels (Fig. 3). At the socio-political level, modifications were influenced by local, state, and federal regulations that impacted the ability to provide in-person care. In addition, waivers granted by insurance providers opened the possibility that some services provided by physical or behavioral health therapists using telehealth were newly reimbursable [42]. Organization-level factors included COVID-related staffing shortages and competing demands for provider time that lengthened wait times to initiate treatment. Participant-level factors influencing the modification decisions included recognition that issues around financial concerns, convenience, and motivation were likely impacting treatment initiation pre-COVID. Participants found it burdensome to obtain financial information directly from their insurers. Concerns about personal safety and willingness to receive in-person treatment were additional participant-level considerations that emerged post-COVID.

## Discussion

The COVID pandemic has disrupted a large number of clinical trials across the translational spectrum, a reality that highlights the important issue of how to report modifications that occur in response to extenuating circumstances [43]. This issue, however, did not originate with COVID and will continue to apply after COVID recedes as a disruptive force in society and health care. The implementation of CHIs in pragmatic and CER studies is expected to involve modifications even in the absence of external circumstances like a pandemic, but these modifications are not consistently reported. PCORI recently published methodologic standards for CER studies involving CHIs that clearly support the need to carefully consider and characterize modifications made [7]. The purpose of this report is to describe how modifications were made to the OPTIMIZE trial using FRAME as a comprehensive guide. While this trial is ongoing, explaining how and why modifications were made will assist with the eventual interpretation of our findings and may inform future implementation efforts. We also hope that outlining one team’s approach to unanticipated modifications will spur further discussion of this important topic.

The study team believed the FRAME was well-suited to our goal of characterizing modifications to the OPTIMIZE trial. First, FRAME recognizes that who participates in decisions about modifications is an important consideration. In the context of patient-centered research that is designed to inform end-users, engaging a diverse set of stakeholders in the decision-making to the greatest extent possible is essential. The COVID-related suspension of enrollment in our trial allowed the study team to engage with patients, providers, and health care leaders on our external advisory panel and incorporate their input into the ongoing process of determining our modifications.

Engaging patients on our advisory panel helped clarify some of the reasons for lower-than-expected treatment initiation. Wide variation in insurance coverage for nonpharmacologic treatments for persons with cLBP is well-established [22] and financial concerns can be a barrier to accessing care [44]. Beyond financial considerations, the complexity of navigating the health care system, particularly in the COVID era, is a barrier to treatment attendance, especially for in-person care [45, 46]. Barriers to treatment initiation and persistence due to motivation or ambivalence are described for persons considering CHIs [47, 48] and in our team’s discussions with stakeholders emerged as likely barriers for individuals considering engaging in the OPTIMIZE trial interventions. Motivational interviewing is a counseling style for eliciting behavior change by helping persons to identify and resolve ambivalence about making a change [37]. We decided to use communication techniques grounded in MI because our intent was to help individuals considering enrollment to reflect on their internal motivation and ambivalence around treatment, and support their autonomy in making the best decision about enrollment [49]. We did not train staff to use MI as a counseling strategy aimed at changing behavior, but as a communication strategy to empower individuals to make the best decision about the enrollment. Our goal was not to increase the number of persons who enrolled in the trial, but to enhance the likelihood that those who did enroll would initiate treatment.

As the OPTIMIZE team identified these barriers to treatment, we had considerable discussions with representatives of the funder and stakeholders about appropriate modifications. These discussions focused on balancing considerations around pragmatically reflecting the reality that many persons referred to CHIs chose not to initiate treatment, with concerns that low initiation risked attenuating intervention effects [50]. Pragmatic and CER studies emphasize intention-to-treat principles for the primary analyses of the results with participants analyzed with their randomly assigned group irrespective of compliance [51]. While this approach appropriately reflects the reality that part of a CHI’s effectiveness is the ability and willingness of patients to engage with the intervention, if too few participants actually receive the treatment, it becomes increasingly unlikely that even a large trial will have sufficient statistical power to detect any differences that may exist between treatments [52]. Our team therefore determined that modifications to our recruitment approach and the role of the study staff were appropriate to preserve our ability to detect differences in the effectiveness among the CHIs in the trial. We note that it can be argued that our modifications to the recruitment process might be considered pragmatic in the context of informed decision-making and patient-centered care as we sought to provide more information and assistance to participants [53]. We are aware however that our modifications extend the typical procedures used by health care systems to engage patients with cLBP in decisions about their care and will need to be taken into consideration when interpreting our eventual findings.

Our experience with our modifications to the recruitment process has been positive thus far. One year after resuming enrollment, the rate of treatment initiation into phase I improved from 65 to 71%, and for phase II, improved from 43 to 57%. Changes to the recruitment process and increased treatment initiation have also had a positive impact on rates of obtaining follow-up assessments as treatment discontinuation and loss-to-follow-up are known to be related [54].

An important consideration highlighted by FRAME and consistent with PCORI methodologic standards is the relationship between modifications and intervention fidelity [7, 11]. Fidelity in the context of pragmatic and CER studies, particularly those investigating CHIs, makes an important distinction between core functions, or the fundamental purposes of a CHI, and its forms, or the specific activities used to carry out the core functions [29]. Fidelity-consistent modifications may alter a CHI’s forms, but preserve core functions [6]. Failure to preserve a CHI’s core functions in a pragmatic or CER study risks reducing the uniqueness of the interventions and may make it more difficult to differentiate between CHIs in a study. Ensuring modifications were fidelity-consistent with a key consideration for the study team as well as the study’s funder and stakeholders. Specifically, the ability to preserve the core functions of the PT intervention when using telehealth was a concern.

The study team discussed standardizing delivery (in-person or telehealth) for all CHIs in the trial (PT, CBT, and MORE). This option would have preserved consistency in the form of CHI delivery, which is appealing in more explanatory trials or with less complex interventions [55]. In the OPTIMIZE trial, however, we determined that requiring all CHIs be delivered via telehealth would sacrifice a core function of the PT intervention and diminish the distinctiveness of PT from the other intervention arms. Conversely, requiring all CHIs be delivered in person could run counter to risk mitigation strategies adopted in participating health care systems that were continuing to provide the majority of behavioral health sessions using telehealth. These considerations led our team to allow for adaptations in the form of CHI delivery based on local practices, which helped preserve the CHI core functions to the greatest extent.

Prior to the COVID pandemic, there was a considerable discussion around standards for transparent reporting of modifications of study interventions due to anticipated or unanticipated factors for CER and pragmatic studies [11, 12, 30]. The large number of clinical trials across the translational spectrum that have been disrupted by COVID has increased attention in the broader scientific community on standards for reporting modifications. The CONSORT and SPIRIT Extension for RCTs Revised in Extenuating Circumstance (CONSERVE) statement provides guidance to investigators for reporting trial protocols or results when important modifications were made based on extenuating circumstances [56]. The CONSERVE checklist is consistent with the FRAME components and includes the need to explain why, how, and what types of modifications were made along with who developed and approved the modifications [43]. CONSERVE also makes recommendations for explicit reporting on the use of interim data such as recruitment or adherence rates in making modification decisions [43], which is not explicitly reflected in the FRAME. Accumulating interim data on treatment initiation as part of our data and safety monitoring procedures was very important in alerting our team to a critical issue in the conduct of the trial and the need to make modifications. These data were not evaluated by the treatment group and thus have a low potential to introduce bias [57]. Low treatment initiation was not a concern that arose due to COVID; however, it is likely that treatment initiation would have become a larger problem during successive COVID waves due to staffing fluctuations had modifications not been made.

## Conclusion

Modifications to interventions in clinical trials are common, particularly CER studies evaluating complex interventions, but often unreported. The need for complete and transparent reporting of modifications, both planned and unplanned, has been a topic of increased focus due to the COVID pandemic. Our study team found the FRAME to serve as a useful tool for characterizing modifications made to the OPTIMIZE trial.

## Data Availability

The datasets generated and/or analyzed during the current study are not publicly available because the parent study is ongoing.
